# EVIDENCE-BASED REVIEW & RECOMMENDATIONS FROM THE BRAZILIAN SOCIETY OF SHOULDER AND ELBOW SURGERY: BANKART REPAIR WITH REMPLISSAGE FOR RECURRENT GLENOHUMERAL INSTABILITY WITH BONE LOSS

**DOI:** 10.1590/1413-785220263401e303256

**Published:** 2026-08-21

**Authors:** Leonardo Zanesco, Gustavo de Mello Ribeiro Pinto, Marcus Vinícius Galvão Amaral, João Artur Bonadiman, Kaleu Costa Neri, Flávio de Oliveira França, Marcelo Costa de Oliveira Campos, Eduardo Angeli Malavolta

**Affiliations:** 1 Universidade de Sao Paulo, Faculdade de Medicina, Hospital das Clinicas HCFMUSP, Sao Paulo, SP, Brazil.; 2 Hcor, Sao Paulo, SP, Brazil.; 3 Universidade do Estado do Rio de Janeiro (UERJ), Rio de Janeiro, RJ, Brazil.; 4 Instituto Nacional de Traumatologia e Ortopedia Jamil Haddad, Centro de Cirurgia do Ombro e Cotovelo, Rio de Janeiro, RJ, Brazil.; 5 Hospital Sao Vicente de Paulo, Instituto de Ortopedia e Traumatologia, Passo Fundo, RS, Brazil.; 6 Policlinica Medica do Corpo de Bombeiros Militar do Distrito Federal, Brasilia, DF, Brazil.; 7 Hospital Ortopedico de Belo Horizonte, Belo Horizonte, MG, Brazil.

**Keywords:** Shoulder Dislocation, Glenohumeral Joint, Joint Instability, Bankart Lesions, Hill Sachs Lesions, Arthroscopy, Luxação do Ombro, Articulação Glenoumeral, Instabilidade Articular, Lesões de Bankart, Lesão de Hill-Sachs, Artroscopia

## Abstract

**Introduction:**

Recurrent anterior glenohumeral instability with "on-track" Hill-Sachs lesions and subcritical glenoid bone loss remains a therapeutic challenge, with considerable failure rates following isolated arthroscopic Bankart repair (BR). The addition of the Remplissage procedure (BR-R) has been proposed to improve stability and shoulder function, however, its effectiveness in "on-track" lesions remains uncertain.

**Methods:**

An evidence-based review was conducted in PubMed, Embase, Cochrane Library, and BVS (search up to August 2025), including comparative clinical studies that evaluated BR-R versus isolated BR in patients with anterior instability, on-track Hill-Sachs lesions, and partial glenoid bone loss. Primary outcomes were recurrence of dislocation and shoulder function; secondary outcomes included residual apprehension, reoperation, and return to sport.

**Results:**

Four studies met the inclusion criteria (one systematic review of six cohort studies and three additional cohort studies; 537 patients in total). Across studies, BR-R showed lower recurrence rates (0–7.7% vs. 3.5–30%) and better functional scores (WOSI, SSV, SANE, ASES, ROWE), higher return-to-sport rates at preinjury level in athletes, and less residual apprehension, without clear increase in complications. Overall certainty of evidence was very low.

**Conclusions:**

Very low-quality evidence suggests that the addition of Remplissage to arthroscopic Bankart repair may improve stability and functional outcomes compared with isolated Bankart repair in patients with on-track bone loss. ***Level of evidence II; Systematic review.***

## RESUMO

**Introdução:**

A instabilidade glenoumeral anterior recidivante com lesões de Hill-Sachs "on-track" e perda óssea glenoidal subcrítica permanece desafiadora, e as taxas de falha após o reparo artroscópico isolado de Bankart (RB) podem ser significativas. A adição do procedimento de Remplissage (RB-R) foi proposta para melhorar a estabilidade e a função do ombro, porém sua efetividade em lesões "on-track" ainda não está completamente estabelecida.

**Métodos:**

Foi realizada uma revisão baseada em evidência nas bases PubMed, Embase, Cochrane Library e BVS (busca até agosto de 2025), incluindo estudos clínicos comparativos que avaliaram RB-R versus RB isolado em pacientes com instabilidade anterior, lesões de Hill-Sachs "on-track" e perda óssea glenoidal parcial. Os desfechos primários foram recidiva da luxação e função do ombro; desfechos secundários incluíram apreensão residual, reoperação e retorno ao esporte.

**Resultados:**

Quatro estudos preencheram os critérios de inclusão (uma revisão sistemática de seis estudos de coorte e três estudos de coorte adicionais; total de 537 pacientes). Em conjunto, os estudos mostraram que o RB-R apresenta menores taxas de recidiva (0–7,7% vs. 3,5–30%) e melhores escores funcionais (WOSI, SSV, SANE, ASES, ROWE), maior taxa de retorno ao esporte no nível pré-lesão em atletas e menor apreensão residual, sem aumento claro nas complicações. A certeza global da evidência foi classificada como muito baixa.

**Conclusão:**

A evidência, embora de muito baixa qualidade, sugere que a adição do Remplissage ao reparo artroscópico de Bankart melhora a estabilidade e os desfechos funcionais em comparação ao reparo isolado de Bankart em pacientes com perda óssea do tipo "on-track". ***Nível de Evidência II; Revisão sistemática.***

## RECOMMENDATION

There is evidence, although of very low quality, that adding the Remplissage procedure to Bankart repair is superior to isolated Bankart repair, resulting in lower recurrence rates and better shoulder function (Level of Evidence III) in the treatment of lesions with "on-track" bone loss. However, studies with greater methodological rigor are needed to clarify this issue further.

The SBCOC Consensus Project recommends that anterior instabilities with "on-track" bone loss be individually evaluated regarding the risk of recurrence. In these cases, adding Remplissage to arthroscopic Bankart repair may provide better functional outcomes, lower recurrence rates, and reduced residual apprehension, especially in patients with higher functional demands or lesions close to the critical threshold of bone loss.

## INTRODUCTION

Glenohumeral dislocation is the most frequent dislocation in the human body, accounting for approximately 50% of all joint dislocations. The annual incidence is estimated at 23.9 per 100,000 people, with traumatic anterior dislocation being the most prevalent form, especially in young and active individuals such as athletes and military personnel.^1,2^ After a first episode of traumatic anterior dislocation, the risk of recurrence is particularly high in male patients under 20 years of age, and may exceed 70% in this population.^1,3^

Traditionally, in the absence of evident risk factors for recurrence—such as significant bone defects—conservative treatment is usually the initial approach after the first dislocation episode.^1,3,4^ However, in the setting of recurrent instability or relevant bone loss, surgical treatment is indicated. The most commonly used techniques are arthroscopic Bankart repair and bone-block procedures, with the Latarjet technique being the most widely performed within this latter group.^2,5,6^

The "glenoid track" concept, described by Yamamoto et al. in 2007^7^, represented a significant advance in the understanding of glenohumeral instability, especially in cases with bipolar bone lesions.^8^ According to this concept, the extent of the Hill-Sachs lesion and its relationship to the remaining glenoid width determine whether the lesion is "on-track" (within the glenoid contact zone during motion) or "off-track" (outside the contact zone), with a risk of engaging and recurrence.^5,7,9^ This classification allows for more accurate patient stratification, helping to guide the choice of the most appropriate surgical procedure.^5^

However, even in cases classified as "on-track" but with significant bone loss or a large Hill-Sachs lesion, isolated Bankart repair (BR) has been associated with considerable failure rates.^5,6,9^ In this context, performing a Bankart repair combined with Remplissage (BR-R)—tenodesis of the infraspinatus tendon to fill the humeral head defect^10^—aimed at filling the Hill-Sachs lesion and reducing the risk of engaging and recurrent instability, appears to be a logical strategy. The present consensus aims to review the current literature to assess whether adding Remplissage to arthroscopic Bankart repair in patients with "on-track" lesions and partial bone loss can improve clinical outcomes and reduce the failure rate of joint stabilization.

This evidence review was developed within a collaborative initiative between Cochrane and the Brazilian Society of Shoulder and Elbow Surgery (SBCOC). The aim of this partnership is to synthesize the best available evidence using rigorous, transparent methods and to translate it into clear, practical recommendations for clinical practice

### Research Question

In adults with recurrent anterior glenohumeral instability, 'on-track' Hill-Sachs lesions, and subcritical (partial) glenoid bone loss, which approach is more effective — arthroscopic Bankart repair combined with remplissage (BR-R) or isolated Bankart repair (BR) — regarding recurrence of dislocation and shoulder function?"

## METHODS

- Databases searched: PubMed, Embase, Cochrane Library, and BVS.
- Descriptors used: "Adolescent", "Adult", "Arthroscopy* / methods", "Bankart Lesions / surgery", "Double-Blind Method", "Female", "Follow-Up Studies", "Humans", "Joint Instability* / surgery", "Male", "Middle Aged", "Recurrence*", "Reoperation* / statistics & numerical data", "Shoulder Dislocation / surgery", "Shoulder Joint / surgery", and "Young Adult". (see Appendix 1).
- Search period: Up to August 2025.
- Study selection: according to the highest level of evidence, prioritizing systematic reviews (SRs) of the literature with good methodological quality and up-to-date evidence. In the absence of SRs, randomized controlled trials (RCTs) were considered, followed by observational studies and, finally, case series.

## RESULTS

**Figure 1:**
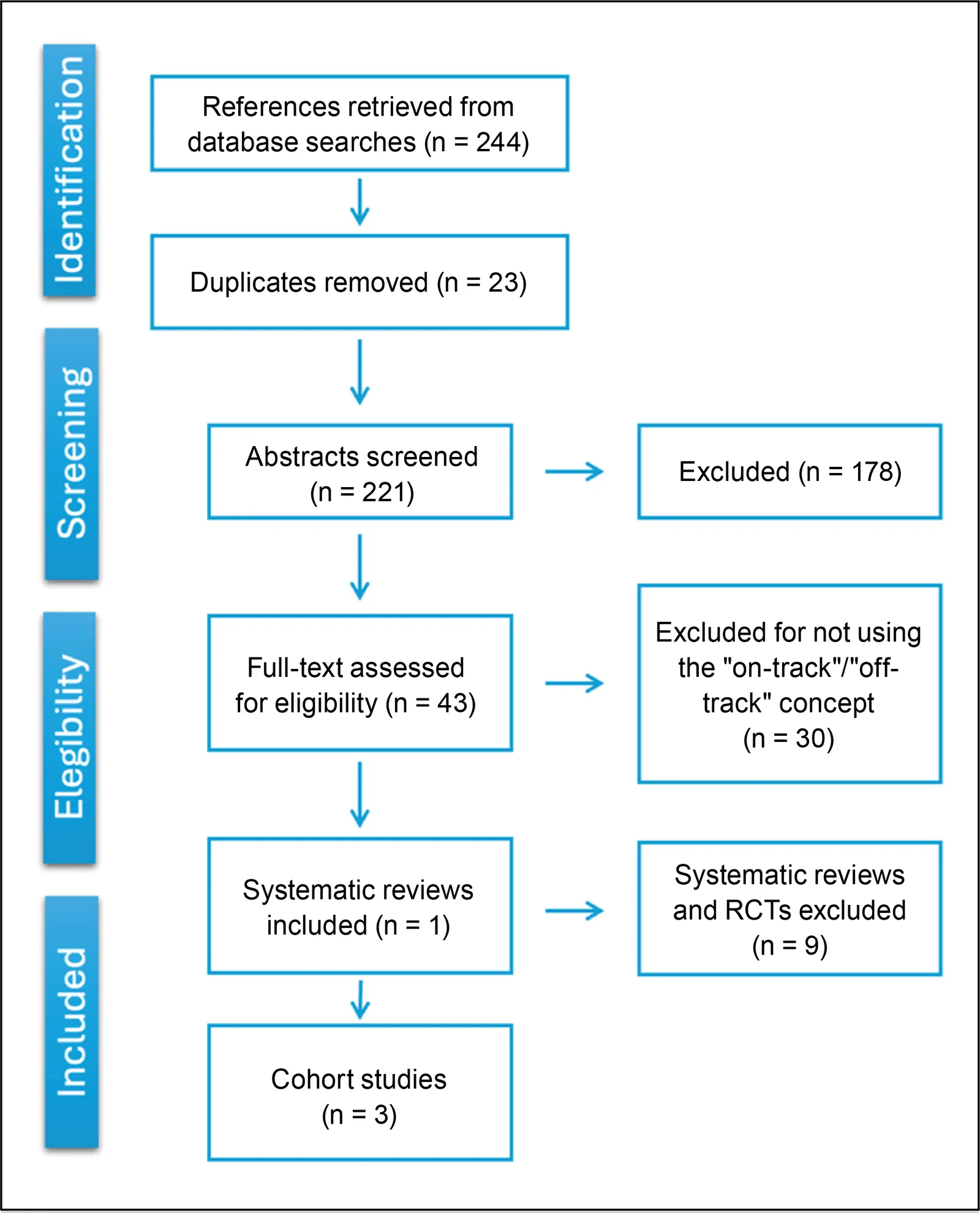
Flow diagram of the literature search.

The search identified 244 references (55 in PubMed, 126 in Embase, 36 in the Cochrane Library, and 27 in BVS). Forty-three full-text articles were selected for evaluation, of which 30 were excluded for not incorporating the glenoid track concept (they either did not include or report on-track lesions, did not classify lesions as on-track or off-track, or included off-track lesions in their samples)^11–40^, and 9 were excluded for not comparing the methods under investigation.^41–49^ Thus, 4 studies were included in the scope of this review.^50–53^ (Figure 1)

### Included studies

Few scientific studies were identified, most of them retrospective cohort studies. In a multicenter cohort study conducted by Lin et al.^50^ (2023), 183 patients aged 14 to 50 years were evaluated with a minimum follow-up of two years, 56 in the BR-R group and 127 in the BR group. All had "on-track" lesions, without glenoid bone loss greater than 15% or other associated comorbidities. The BR-R group had only one recurrence (1.8%) and no reoperations. In the BR group, 14 patients (11.0%) experienced a new dislocation (p = 0.040) and 8 (6.3%) required reoperation (p = 0.11). In addition, the high-risk BR-R subgroup showed significantly better scores on the Western Ontario Shoulder Instability Index (p = 0.008) and Subjective Shoulder Value (p = 0.001) compared with the control group.

In the systematic review by Villarreal et al.^51^ (2025), six level III studies were evaluated, totaling 537 participants. Of these, 202 underwent the combined technique and 335 underwent isolated Bankart repair. After a mean follow-up of 35 months, recurrence rates ranged from 0% to 7.7% in the Remplissage-plus-Bankart group and from 3.5% to 30% in the isolated Bankart group, corresponding to a 78% relative risk reduction (OR 0.22; 95% CI 0.09–0.53).

In the study by Kirac et al.^52^ (2023), involving 64 professional overhead athletes (basketball, volleyball, and handball) with "on-track" lesions and subcritical glenoid bone loss, adding Remplissage to arthroscopic Bankart repair resulted in better functional scores (SANE, ASES, and ROWE), higher return-to-sport rates at the preinjury level, and a lower frequency of subjective apprehension during sports practice compared with isolated Bankart repair. Notably, there were three recurrences in the BR group and none in the BR-R group, reinforcing the effectiveness of Remplissage in preventing recurrent instability even in "on-track" lesions.

Finally, in the study by Yu et al.^53^ (2023), adding Remplissage to arthroscopic Bankart repair resulted in significantly higher ROWE scores and a lower frequency of residual apprehension.

The general characteristics of the evaluated studies and the quality of evidence according to the GRADE system^54,55^ are presented in Tables 1 and 2, respectively.

**Table 1:** Characteristics of the included studies.

Studies	Study type	Included studies (Designs)	Sample size	Published protocol
Lin et al (2023)	Cohort Study	- -	183	No
Villarreal et al (2025)	Systematic Review	6 (6 Cohorts)	537	No
Kirac et al (2023)	Cohort Study	- -	64	No
Yu et al (2023)	Cohort Study	- -	53	No

**Table 2:** Quality of evidence (GRADE): Bankart repair vs. Bankart repair associated with the Remplissage procedure.

Outcome	Studies (n)	Evidence quality
Recurrent Dislocation	6 studies (537)	Very low
Shoulder Function	6 studies (537)	Very low

The GRADE tool (Grading of Recommendations Assessment, Development and Evaluation) is used to appraise and measure the quality of evidence. It is grounded in five domains: risk of bias, imprecision, inconsistency, indirectness of outcomes, and publication bias. Based on this framework, the quality of evidence is classified into four levels: very low, low, moderate, and high ^54,55^.

## CONCLUSION

There is very low-quality evidence showing that Bankart repair combined with Remplissage is superior to isolated Bankart repair in terms of recurrent dislocation and functional outcomes.

## Data Availability

The data underlying this study are available within the article.

## Appendix 1 Database search strategies.

**Table:**

**PUBMED**
#1 "Shoulder Joint"[Mesh] OR (Joint, Shoulder) OR (Joints, Shoulder) OR (Shoulder Joints) OR (Glenohumeral Joint) OR (Glenohumeral Joints) OR (Joint, Glenohumeral) OR (Joints, Glenohumeral) OR (Glenoid Labrum) OR (Labrum, Glenoid) OR (Shoulder Dislocation) OR (Joint Instability)
#2 "Bankart Lesions"[Mesh] OR (Lesions, Bankart) OR (Bankart Lesion) OR (Lesion, Bankart) OR (Bankart Tears) OR (Tears, Bankart) OR (Hill-Sachs Lesion) OR (Hill Sachs Lesion) OR (Lesion, Hill-Sachs) OR (Hill-Sachs Lesions) OR (Hill Sachs Lesions) OR (Lesions, Hill-Sachs) OR (Bankart Fractures) OR (Fractures, Bankart) OR (Bony Bankart Lesion) OR (Bankart Lesion, Bony) OR (Lesion, Bony Bankart) OR (Bony) OR (Bankart Lesions) OR (Bankart Lesions) OR (, Bony) OR (Lesions, Bony Bankart) OR (Osseous) OR (Bankart Lesions) OR (Bankart Lesions) OR (, Osseous) OR (Lesions, Osseous Bankart) OR (Osseous Bankart Lesion) OR (Bankart Lesion, Osseous) OR (Lesion, Osseous Bankart)
#3 "Arthroscopy"[Mesh] OR (Arthroscopies) OR (Arthroscopic Surgical Procedures) OR (Arthroscopic Surgical Procedure) OR (Procedure, Arthroscopic Surgical) OR (Procedures, Arthroscopic Surgical) OR (Surgical Procedure, Arthroscopic) OR (Arthroscopic Surgery) OR (Arthroscopic Surgeries) OR (Surgeries, Arthroscopic) OR (Surgery, Arthroscopic) OR (Surgical Procedures, Arthroscopic)
#4 (Repair Bankart) OR(Arthroscopic Bankart Repair) OR (Remplissage Procedure) OR (Arthroscopic Bankart Repair With Remplissage) OR (Bankart Repair MORE Remplissage) OR (Bankart Repair AND Remplissage)
#5 Recurrence* OR Reoperation
#6 #1 AND #2 AND #3 AND #4AND #5

**Table:**

**COCHRANE**
#1 (Shoulder Joint) OR (Joint, Shoulder) OR (Joints, Shoulder) OR (Shoulder Joints) OR (Glenohumeral Joint) OR (Glenohumeral Joints) OR (Joint, Glenohumeral) OR (Joints, Glenohumeral) OR (Glenoid Labrum) OR (Labrum, Glenoid) OR (Shoulder Dislocation) OR (Joint Instability)
#2 (Bankart Lesions) OR (Lesions, Bankart) OR (Bankart Lesion) OR (Lesion, Bankart) OR (Bankart Tears) OR (Tears, Bankart) OR (Hill-Sachs Lesion) OR (Hill Sachs Lesion) OR (Lesion, Hill-Sachs) OR (Hill-Sachs Lesions) OR (Hill Sachs Lesions) OR (Lesions, Hill-Sachs) OR (Bankart Fractures) OR (Fractures, Bankart) OR (Bony Bankart Lesion) OR (Bankart Lesion, Bony) OR (Lesion, Bony Bankart) OR (Bony) OR (Bankart Lesions) OR (Bankart Lesions) OR (, Bony) OR (Lesions, Bony Bankart) OR (Osseous) OR (Bankart Lesions) OR (Bankart Lesions) OR (, Osseous) OR (Lesions, Osseous Bankart) OR (Osseous Bankart Lesion) OR (Bankart Lesion, Osseous) OR (Lesion, Osseous Bankart)
#3(Arthroscopy) OR (Arthroscopies) OR (Arthroscopic Surgical Procedures) OR (Arthroscopic Surgical Procedure) OR (Procedure, Arthroscopic Surgical) OR (Procedures, Arthroscopic Surgical) OR (Surgical Procedure, Arthroscopic) OR (Arthroscopic Surgery) OR (Arthroscopic Surgeries) OR (Surgeries, Arthroscopic) OR (Surgery, Arthroscopic) OR (Surgical Procedures, Arthroscopic)
#4 (Repair Bankart) OR (Arthroscopic Bankart Repair) OR (Remplissage Procedure) OR (Arthroscopic Bankart Repair With Remplissage) OR (Bankart Repair MORE Remplissage) OR (Bankart Repair AND Remplissage)
#5 Recurrence* OR Reoperation
#6 #1 AND #2 AND #3 AND #4 AND #5

**Table:**

**EMBASE**
#1 'shoulder dislocation'/exp OR 'dislocation, shoulder' OR 'humeroscapular luxation' OR 'scapulohumeral luxation' OR 'shoulder dislocation' OR 'shoulder joint dislocation' OR 'shoulder luxation'
#2 'bankart lesion'/exp OR 'bankart fracture' OR 'bankart fractures' OR 'bankart lesion' OR 'bankart lesions' OR 'bankart tear' OR 'hill-sachs lesion' OR 'bony bankart lesion' OR 'osseous bankart lesion'
#3 'arthroscopy'/exp OR 'arthro-endoscopy' OR 'arthroendoscopy' OR 'arthroscopic exam' OR 'arthroscopic examination' OR 'arthroscopic procedure' OR 'arthroscopic procedures' OR 'arthroscopy'
#4 (Repair Bankart) OR (Arthroscopic Bankart Repair) OR (Remplissage Procedure) OR (Arthroscopic Bankart Repair With Remplissage) OR (Bankart Repair MORE Remplissage) OR (Bankart Repair AND Remplissage)
#5 Recurrence* OR Reoperation
#6 #1 AND #2 AND #3 AND #4 AND #5

**Table:**

**PORTAL REGIONAL BVS**
#1 MH:"Articulação do Ombro" OR (Shoulder Joint) OR (Articulação Glenoumeral) OR (Articulações dos Ombros) OR (Ligamento Glenoide Umeral) OR (Lábio Glenoidal) OR MH:A02.835.583.748$
#2MH:"Lesões de Bankart" OR (Bankart Lesions) OR (Fraturas Bankart) OR (Fraturas de Bankart) OR (Lacerações Bankart) OR (Lacerações de Bankart) OR (Lesão Bankart) OR (Lesão de Bankart) OR (Lesão de Hill-Sachs) OR (Lesão Óssea de Bankart) OR (Hill-Sachs) OR (Lesões Ósseas de Bankart) OR (Rupturas Bankart) OR (Rupturas de Bankart) OR MH:C26.404.625.500$ OR MH:C26.803.250.500$
#3MH:"Artroscopia" OR (Arthroscopy) OR (Procedimentos Cirúrgicos Artroscópicos) OR MH:E01.370.388.250.070$ OR MH:E04.502.250.070$ OR MH:E04.555.113$
#4 (Repair Bankart) OR (Arthroscopic Bankart Repair) OR (Remplissage Procedure) OR (Arthroscopic Bankart Repair With Remplissage) OR (Bankart Repair MORE Remplissage) OR (Bankart Repair AND Remplissage)
#5 #1 AND #2 AND #3 AND #4
